# Sulfur Amino Acid Status Controls Selenium Methylation in Pseudomonas tolaasii: Identification of a Novel Metabolite from Promiscuous Enzyme Reactions

**DOI:** 10.1128/AEM.00104-21

**Published:** 2021-05-26

**Authors:** Ying Liu, Sebastian Hedwig, Andreas Schäffer, Markus Lenz, Mathieu Martinez

**Affiliations:** a Institute for Ecopreneurship, School of Life Sciences, University of Applied Sciences and Arts Northwestern Switzerland, Muttenz, Switzerland; b Institute for Environmental Research, RWTH Aachen University, Aachen, Germany; c University of Basel, Department of Chemistry, Basel, Switzerland; d Sub-Department of Environmental Technology, Wageningen University, Wageningen, the Netherlands; Chinese Academy of Sciences

**Keywords:** selenium cycling, selenium deficiency, selenium fate, trace element fate, atmospheric selenium

## Abstract

Selenium (Se) deficiency affects many millions of people worldwide, and the volatilization of methylated Se species to the atmosphere may prevent Se from entering the food chain. Despite the extent of Se deficiency, little is known about fluxes in volatile Se species and their temporal and spatial variation in the environment, giving rise to uncertainty in atmospheric transport models. To systematically determine fluxes, one can rely on laboratory microcosm experiments to quantify Se volatilization in different conditions. Here, it is demonstrated that the sulfur (S) status of bacteria crucially determines the amount of Se volatilized. Solid-phase microextraction gas chromatography mass spectrometry showed that Pseudomonas tolaasii efficiently and rapidly (92% in 18 h) volatilized Se to dimethyl diselenide and dimethyl selenyl sulfide through promiscuous enzymatic reactions with the S metabolism. However, when the cells were supplemented with cystine (but not methionine), a major proportion of the Se (∼48%) was channeled to thus-far-unknown, nonvolatile Se compounds at the expense of the previously formed dimethyl diselenide and dimethyl selenyl sulfide (accounting for <4% of total Se). Ion chromatography and solid-phase extraction were used to isolate unknowns, and electrospray ionization ion trap mass spectrometry, electrospray ionization quadrupole time-of-flight mass spectrometry, and microprobe nuclear magnetic resonance spectrometry were used to identify the major unknown as a novel Se metabolite, 2-hydroxy-3-(methylselanyl)propanoic acid. Environmental S concentrations often exceed Se concentrations by orders of magnitude. This suggests that in fact S status may be a major control of selenium fluxes to the atmosphere.

**IMPORTANCE** Volatilization from soil to the atmosphere is a major driver for Se deficiency. “Bottom-up” models for atmospheric Se transport are based on laboratory experiments quantifying volatile Se compounds. The high Se and low S concentrations in such studies poorly represent the environment. Here, we show that S amino acid status has in fact a decisive effect on the production of volatile Se species in Pseudomonas tolaasii. When the strain was supplemented with S amino acids, a major proportion of the Se was channeled to thus-far-unknown, nonvolatile Se compounds at the expense of volatile compounds. This hierarchical control of the microbial S amino acid status on Se cycling has been thus far neglected. Understanding these interactions—if they occur in the environment—will help to improve atmospheric Se models and thus predict drivers of Se deficiency.

## INTRODUCTION

The trace element selenium (Se) is essential for human and animal health. Se is known to be a “double-edged sword” element, maintaining one of the narrowest ranges between dietary deficiency (<40 μg/day) and toxic levels (>400 μg/day) in humans (1). Whereas Se toxicity due to bioaccumulation and biomagnification is a local problem (for example, see references 2 and 3), there are many Se-deficient regions worldwide (4), and an estimated 0.5 to 1 billion people may be negatively affected (5). Therefore, understanding the physicochemical and biological processes underlying Se environmental cycling is of the utmost importance.

Volatilization may decrease the available Se in soil and prevent its entry into the food chain, thus worsening Se deficiency. In the atmosphere, transported Se is associated with particulate matter, and subsequent atmospheric deposition may increase Se contamination in surface environments (6, 7). Following early studies (reviewed in references 8–10), biological methylation and subsequent volatilization are known to be major sources of emission to the atmosphere (3, 9, 11). Volatile methylated species can be formed by terrestrial plants (12), fungi (13), soil microbes, or marine algae (14), although the discussion on the prevalence of marine over terrestrial sources continues (for example, see references 11 and 15 and references therein). Despite advances in quantifying atmospheric fluxes, it has been stated that the data available to date are too sparse to establish a reliable flux estimate for Se volatilization from land, which limits the ability to predict volatilized-Se fluxes quantitatively (15).

The lack of reliable quantitative data on biomethylation for most natural (Se-depleted) systems stems from the fact that, historically, experiments focused on applying volatilization for the bioremediation of Se-contaminated soils and sediments or wastewaters concentrated on Se or simply focused on amending laboratory microcosms with Se to simulate the latter (16–23). Therefore, most studies of Se volatilization were performed using Se concentrations 10^3^- to 10^6^-fold higher than those commonly found in aqueous environmental media in the nanogram-per-liter to microgram-per-liter range. Se methylation is mostly regarded as an active detoxification strategy (reference 24 and references therein), because volatilization decreases aqueous Se concentrations and the intracellular Se content. However, some studies found Se volatilization to still be efficient even at trace (nanogram-per-liter) aqueous Se concentrations (25), which are unlikely to induce specific Se detoxification enzymes.

Enzyme promiscuity refers to the ability of an enzyme to catalyze a fortuitous side reaction in addition to its main reaction, and most enzymes can promiscuously catalyze reactions other than those for which they evolved (26). The structural analogy of major Se and sulfur (S) species (namely, selenite/sulfite and selenate/sulfate) shared oxidation states and functional group types may suggest that Se at trace concentrations is in fact volatilized by promiscuous enzymatic reactions of the S metabolism (27), despite their different biogeochemistry. Therefore, Se methylation may be controlled on different levels. First, on the enzymatic level, the structural competition for the active site of the S cycling enzyme determines how much Se is “spuriously” cocycled. Second, the overall S status of the cell determines the expression of anabolic or catabolic enzymes and thus the pathways through which Se can be cycled.

Therefore, this interplay of S status and Se methylation was studied using incubations of a *Pseudomonas* strain, a common model for the volatilization of these elements (28–31). The S status was influenced by the supplementation of two S sources, namely, methionine (Met) and cystine (Cy; disulfide dimer of cysteine). Se and S methylation was quantified by solid-phase microextraction-gas chromatography-mass spectrometry (SPME-GC-MS) measurements. An unknown, nonvolatile Se species that accumulated was identified by a combination of ion chromatography triple-quadrupole inductively coupled plasma mass spectrometry (IC-QqQ-ICP-MS), electrospray ionization ion trap mass spectrometry (ESI-MS/MS), electrospray ionization quadrupole time-of-flight mass spectrometry (ESI-Q-TOF-MS) and microprobe nuclear magnetic resonance (NMR) spectroscopy.

## RESULTS

### Transformation of selenite by Pseudomonas tolaasii.

**FIG 1:**
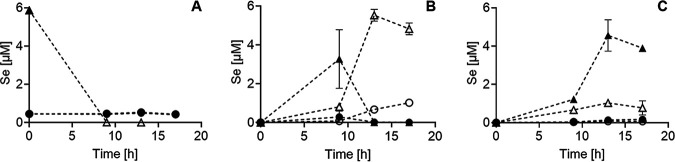
Conversion of selenium in Met+Cy-supplemented (filled symbols) and control (empty symbols) *Pseudomonas* cultures in terms of selenium oxyanions (A) (triangles, selenite; circles, selenate), volatile methylated Se (B) (triangles, DMDSe; circles, DMSeS), and unknown metabolites (C) (triangles, metabolite 1; circles, metabolite 2). Note that no selenite was detected in Met+Cy-supplemented cultures during incubation and that in panel A, the filled and empty triangles are overlaid at 0.

**FIG 2:**
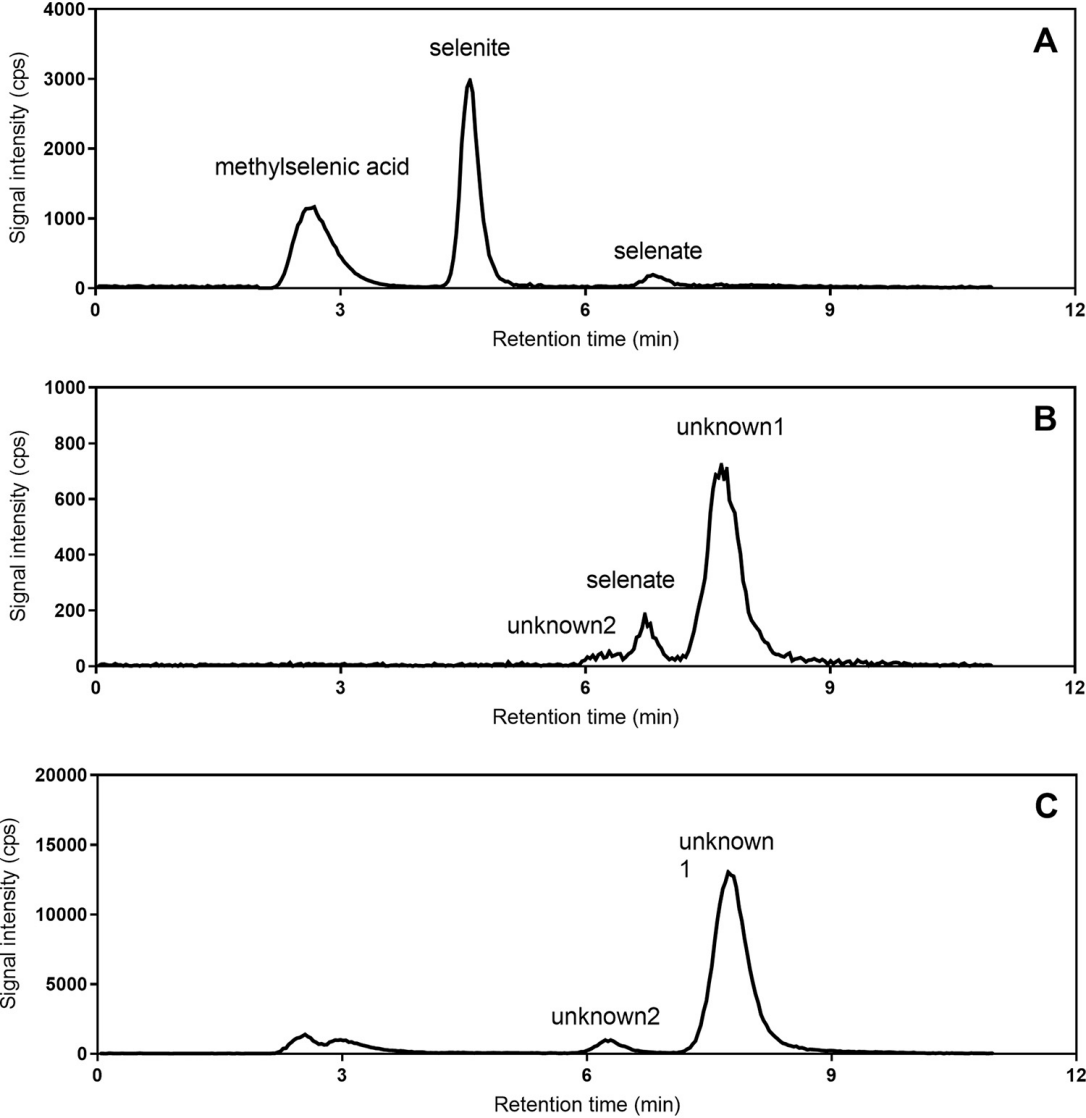
Separation of selenium species by IC-ICP-QqQ (mass shift mode using signal ^78^Se^16^O^+^) of (A) a mix of standards of methylselenic acid, selenate, and selenite; (B) supernatant of the *Pseudomonas* culture supplemented with methionine and cystine; (C) SPE eluates of the concentrated supernatant.

Selenite was rapidly depleted (<10 h) from the medium in all experiments (Fig. 1A). In controls (i.e., not receiving S amino acid supplementation), selenite was almost quantitatively converted to volatile methylated species in the form of dimethyl diselenide (DMDSe) (4.8 μM) and dimethyl selenosulfide (DMSeS) (1.0 μM) (Fig. 1B). Not considering the minor amount of selenate (<0.5 μM; ∼8%) that was found in all experiments as a result of oxidation of the selenite standard, conversion efficiency to volatile methylated species was as high as ∼98% (based on ∼5.9 μM selenite initially). In cultures supplemented with Met and Cy, volatile methylated species were detected only at 9 h (Fig. 1B) and at lower concentrations (3.3 μM DMDSe plus 0.3 μM DMSeS) than in the control. In contrast to the control, both DMDSe and DMSeS disappeared from the solution during further incubation (∼0.01 μM each) (Fig. 1B). At the same time, two unknown peaks (unknown1 and unknown2) in IC-QqQ-ICP-MS arose (Fig. 2B) that represented the major species upon termination of the experiment (3.9 μM and 0.2 μM, respectively) (Fig. 1C). Not considering the minor amounts of selenate, these unknowns species corresponded to ∼70% of all Se.

**FIG 3:**
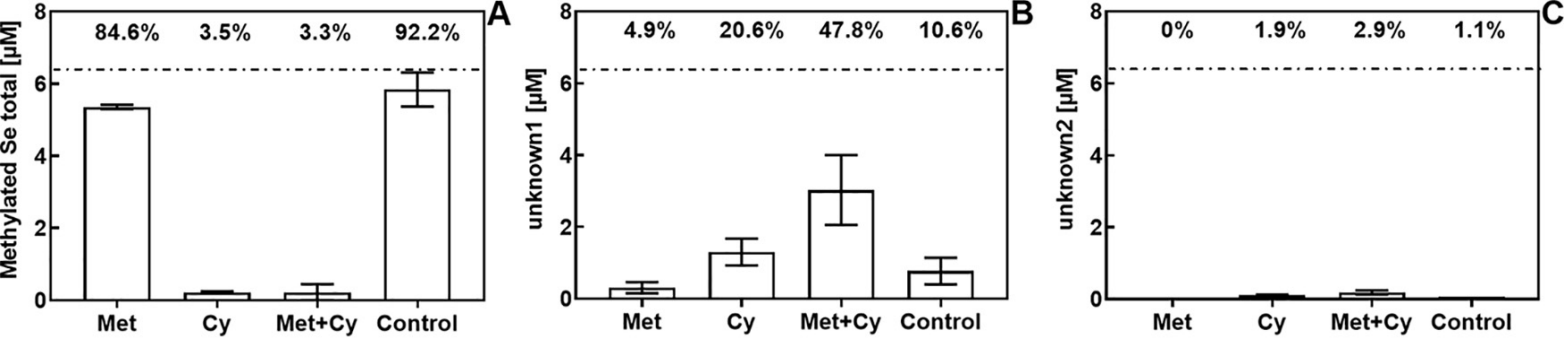
Conversion of selenite to volatile methylated species (A), metabolite 1 (B), and metabolite 2 (C) in *Pseudomonas* cultures at stationary phase (∼18 h) supplemented with methionine alone (Met), cystine alone (Cy), and methionine and cystine (Met+Cy) and without supplementation (control).

To elucidate a possible pathway of formation, Met and Cy were also supplemented as single compounds. An addition of Met and no S-amino acid supplementation (control) resulted in a virtually quantitative (84.6% and 92.2%, respectively) conversion of selenite to volatile methylated species (DMDSe and DMSeS) (Fig. 3A) during the stationary phase (18 h). In contrast, the addition of Cy led to the formation of the unknown metabolites, both as a single supplement of Cy (20.6% of total Se) and in a mixture with Met (47.8% of total Se) (Fig. 3B). Unknown1, eluting at 7.7 min, was present at concentrations between 13 (Cy) and 15 (Met+Cy) times higher than unknown2 (Fig. 3C).

### Purification and concentration of unknown Se species by IC-QqQ-ICP-MS and SPE.

Both unknown1 and unknown2 eluted between sulfate and phosphate (detected by conductivity; data not shown); therefore, the unknowns were assumed to be acidic selenocompounds. For purification and concentration, solid-phase extraction (SPE) was performed on an Oasis HLB cartridge. The culture medium supernatants were at neutral pH, at which the unknowns were not retained on the sorbent of the cartridge (shown by QqQ-ICP-MS; data not shown). A pH of 3 was determined to be suitable to perform SPE, and unknowns were concentrated after NH_3_·H_2_O elution by a factor of ∼20. At the same time, selenate (compare Fig. 2B and C), sulfate, phosphate, and chloride were removed (IC conductivity chromatogram not shown).

### Mass-spectrometric investigation of the unknown Se species.

**FIG 4:**
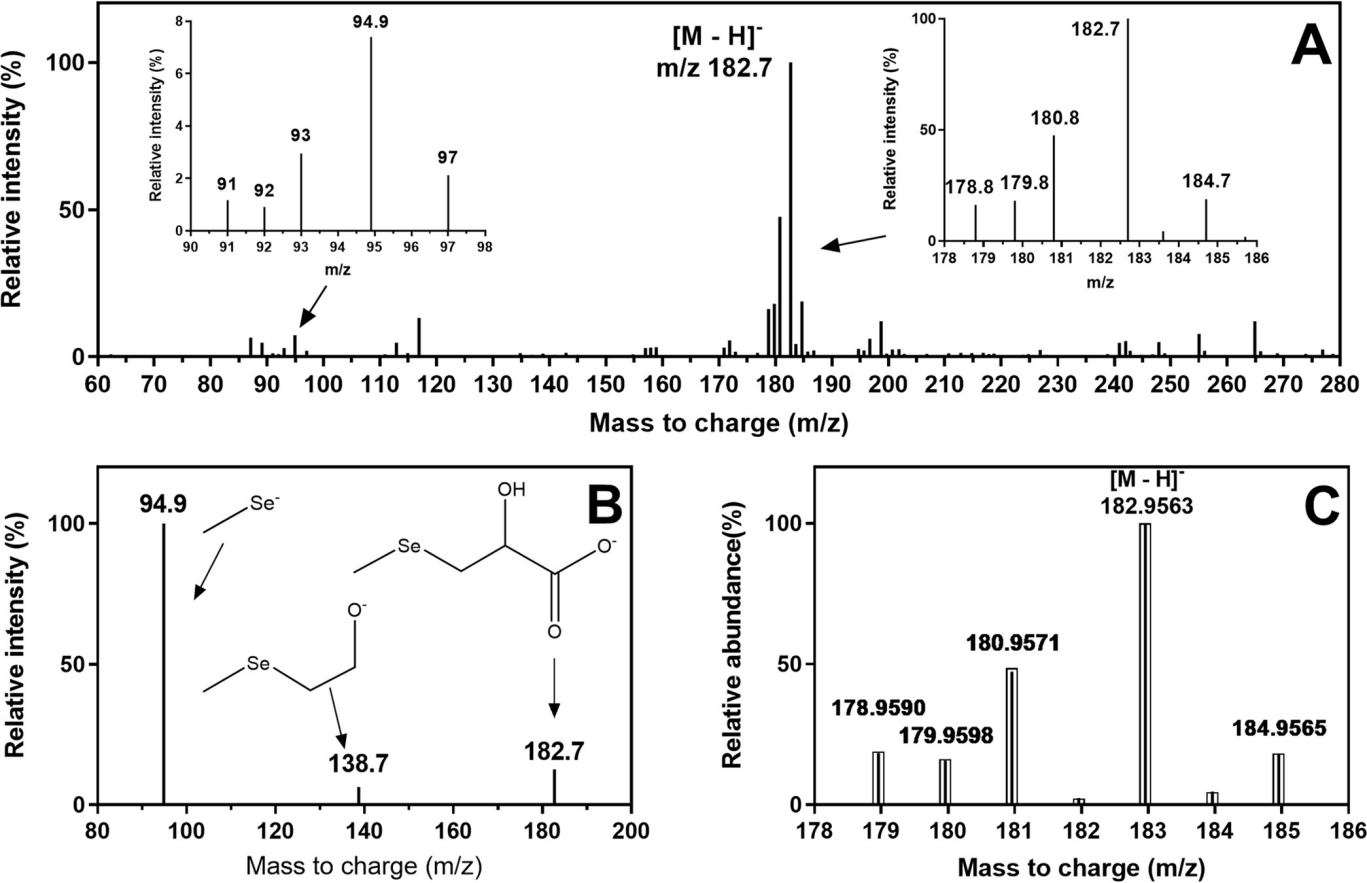
Mass-spectrometric analysis of the collected fraction corresponding to unknown1. (A) ESI-ion trap-MS analysis via full mass scan and zoom on selected mass spectra (insets) demonstrating isotopic pattern of one Se atom. (B) ESI-ion trap-MS analysis via MS^2^ fragmentation of *m/z* 183. (C) ESI-Q-TOF-MS high-resolution mass spectra in negative mode (bars) and calculated mass distribution of [(C_4_H_8_O_3_Se) − H]^−^ (boxes).

To identify the unknown selenocompounds, an ESI-ion trap-MS analysis in negative mode was performed. The full-scan mass spectra of unknown1 showing the isotope patterns of monomeric Se were observed at around *m/z* 183 and 95 (Fig. 4A). The ion *m/z* 183 had the highest abundance and was postulated to be the [M − H]^−^ molecular ion of unknown1. Fragmentation of the ion at *m/z* 183 revealed *m/z* 139 and 95 (Fig. 4B). The mass loss of 44 from 183 to 139 was attributed to the presence of one carboxyl group in the compound. In addition, ion 95 with a monomeric Se isotope pattern indicated the presence of one methylselenol group (CH_3_Se-R) (Fig. 4A). Even with an SPE treatment, unknown2 still had a concentration too low to yield reliable ESI-MS signals and was omitted from further studies.

Q-TOF-MS was then used to acquire the accurate mass of unknown1 as 182.9563 for the molecular ion [M − H]^−^ (Fig. 4C). This mass was in accordance with the molecular formula C_4_H_8_O_3_Se. In addition, the calculated theoretical mass distribution of C_4_H_8_O_3_Se (Fig. 4C) was consistent with the measured mass of unknown1. Based on the exact mass, 11 selenocompounds with one carboxyl group were proposed (see Fig. S2 in the supplemental material). Despite the correct mass, some candidates (b1 and b2) (see Fig. S2) could be ruled out, as selenols (RSeH) form auto-oxidized dimers (RSeSeR′) having an isotopic pattern of two Se atoms (32). Considering the presence of the methyl-selenol group, the candidate list could be further limited to five compounds (a3 and d1 to d4) (Fig. S2).

### NMR study of unknown1.

The proton NMR spectrum of unknown1 showed four main signals belonging to a single compound (Fig. S3). Around 4.43 ppm, a doublet of doublets (dd) appeared, corresponding to the resonance of a single proton, with two ^3^*J*_H,H_ of 6.8 Hz and 4.1 Hz. At 3.16 ppm and 3.04 ppm, two further doublets of doublets were found as the resonance of one of two diastereotopic methylene protons each. In both cases, a ^2^*J*_H,H_ of 13.0 Hz was determined. Furthermore, ^3^*J*_H,H_ of 4.1 Hz (dd at 3.16 ppm) and 6.8 Hz (dd at 3.04 ppm) were found, matching the coupling constants of the dd at 4.43 ppm. Last, a singlet signal corresponding to the resonance of three methyl-group protons appeared at 2.24 ppm. The coupling between the proton signals was verified by a correlated spectroscopy (COSY) experiment. Based on the NMR results, 2-hydroxy-3-(methylselanyl)propanoic acid (2H3MSePA) appeared as the most likely candidate for unknown1 based on the possible structures determined by ESI-ion trap-MS and Q-TOF-MS (i.e., compound d1) (Fig. S2). The corresponding proton signals of the hydroxy and carboxy groups were invisible due to a fast exchange with the solvent and the relatively low concentration of the sample. Additional signals in the spectrum did not belong to the main compound and could not be attributed to a known impurity.

### Confirmation of 2-hydroxy-3-(methylselanyl)propanoic acid as unknown1.

The identity of 2H3MSePA was confirmed by matching retention times in IC-QqQ-ICP-MS of unknown1 in supernatant with the standard (Fig. S4). In addition, a similar ESI-MS spectrum of the whole compound and a similar ESI-MS/MS fragmentation pattern of mass 183 were obtained for the 2H3MSePA standard and the IC collected fraction corresponding to unknown1 (Fig. S5).

## DISCUSSION

### Impact of S status on Se methylation and tentative mechanism of 2H3MSePA formation.

*Pseudomonas* species have been used as model organisms to study Se volatilization (28–30). Pseudomonas tolaasii is known to produce the volatile S compounds methanethiol and dimethyl disulfide (DMDS) (31), among others, and was shown to produce volatile methylated Se in this study as well. Here, experiments demonstrated that the impact of the S status has a profound impact on Se methylation. Instead of converting all Se into volatile compounds (DMDSe and DMSeS), cultures of *Pseudomonas* channeled most Se into a novel, nonvolatile Se metabolite, 2H3MSePA (Fig. 1). This channeling occurred only when the medium was supplemented with Cy, not Met, where only minor amounts of 2H3MSePA were formed (Fig. 3).

**FIG 5:**
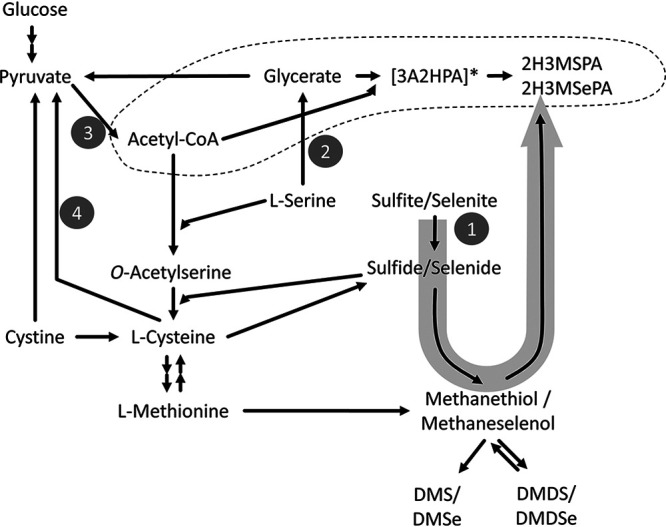
Simplified overview of possible reactions in S metabolism, highlighting proposed promiscuous enzymatic conversion of selenite instead of sulfite (arrow; reaction 1) and tentative mechanism of formation of the novel Se metabolite identified (dashed line). 3A2HPA, 3‐(acetyloxy)‐2‐hydroxypropanoic acid; 2H3MSPA, 2‐hydroxy‐3‐(methylsulfanyl)propanoic acid; 2H3MSePA, 2-hydroxy-3-(methylselanyl)propanoic acid. For details on reactions 1 to 5, see the text.

**FIG 6:**
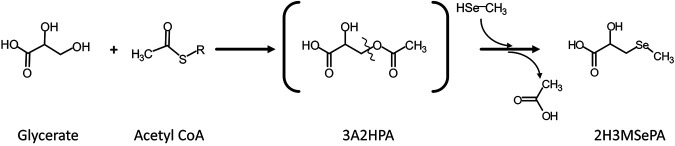
Tentative mechanism of formation of 2‐hydroxy‐3‐(methylselanyl)propanoic acid (2H3MSePA) via 3‐(acetyloxy)‐2‐hydroxypropanoic acid (3A2HPA). Note that the promiscuous enzymatic reaction forming methylselenol is not shown.

It is well known that due to chemical similarities between S and Se, many enzymes involved in S metabolism do not actually discriminate between the two chalcogen elements (33), resulting in promiscuous enzymatic reactions. For instance, Se can enter the bacterial metabolism via the cysteine biosynthetic pathway (34) (reaction 1, Fig. 5). Like S, selenium may be converted to methylated Se species (DMDSe and dimethyl selenide [DMSe]) via methaneselenol (references 35 and 36 and references therein). The back reaction from DMDS to methanethiol is known (37) and can be expected for Se as well. The formation of 2H3MSePA will deplete the methaneselenol pool, thus explaining why DMDSe was formed only transiently (until ∼8 h) in Cy cultures. Although no detailed metabolic study was undertaken, one may speculate about a two-step reaction based on known metabolites and common reactions leading to 2H3MSePA formation (Fig. 6) after a promiscuous enzymatic reaction had formed methaneselenol (reaction 1, Fig. 5): glycerate + acetyl-CoA → [3-acetyloxy-2-hydroxypropanoic acid], and [3-acetyloxy-2-hydroxypropanoic acid] + methylselenol → 2H3MSePA + acetate (where the use of square brackets indicates the speculative nature of the product).

The following observations support the (tentative) proposed mechanisms involved in formation of 2H3MSePA. Glycerate (or glyceric acid) is a central metabolite in, for example, the pentose phosphate pathway, amino acid (glycine, serine, and threonine) metabolism, and glycerolipid metabolism and can be formed from the glucose in the medium used. It is known that the intracellular concentration of cysteine must be tightly controlled due to its reactivity to cofactors and to ensure that its redox homeostasis is retained (38). Therefore, once the cells’ need for cysteine is met (by Cy addition), serine may not be used anymore for its synthesis. Serine can undergo transamination to hydroxypyruvate and further to glycerate (reaction 2, Fig. 5). Met, in contrast, is not redox active or reactive; therefore, it does not require such tight control. It may be degraded to methanethiol and further to DMDS.

Acetyl coenzyme A (acetyl-CoA) is a universal, central intermediate in many metabolic pathways (e.g., citric acid cycle; fatty acid metabolism; valine, leucine, and isoleucine metabolism; etc.). The medium used in this study was glucose based, and glycolysis should be the main mechanism for energy conservation (as in other *Pseudomonas* strains [39]), yielding pyruvate and acetyl-CoA (reaction 3, Fig. 5). Along with glycolysis, acetyl-CoA can be formed during cysteine degradation. Cysteine/cystine are degraded to ammonia, H_2_S, and pyruvate (40, 41) (reaction 4, Fig. 5), which are then oxidized to acetyl-CoA (reaction 3, Fig. 5). The presence of more glycerate (through serine degradation) and more acetyl-CoA (through Cy degradation) possibly explains why more 2H3MSePA is formed under Cy supplementation than with Met.

One should note that the reaction between glycerate and acetyl-CoA has not been directly shown, and the proposed mechanism (Fig. 5) should therefore be considered cautiously. Still, it is a plausible mechanism for forming 3-acetyloxy-2-hydroxypropanoic acid; the transfer of the acetyl group to a terminal hydroxy group of structurally similar compounds is common (e.g., the transfer to l-serine during cysteine biosynthesis). From 3-acetyloxy-2-hydroxypropanoic acid, the second reaction (deacetylation under formation of a R-S-CH_3_ bound or R-Se-CH_3_ bound) may even favor the reaction of Se over S, as methylselenol is a stronger nucleophile than methanethiol (selenolate ions are roughly 1 order of magnitude more nucleophilic than thiolates [27]). Even though the experimental observations in this study were all in line with the above-postulated mechanisms of formation, it should be noted that the detailed mechanism of 2H3MSePA formation remains speculative, and its physiological role (if any) should be elucidated. Alternative modes of formation may be, for instance, via hydroxylation of methyl-selenocysteine, a substrate for bacterial methylation to DMDSe (42). However, as the physiological role and the pathway of methyl-selenocysteine formation are unknown, differential responses to Cy and Met would be speculative as well.

### Implications for atmospheric emission of Se.

Models for transport of atmospheric Se (and other trace metal) can be “top down” (i.e., based on direct measurements of volatile species) or “bottom up” (i.e., extrapolated from laboratory experiments) (reference 43 and references therein). A number of studies have measured volatile Se in marine environments (reference 44 and references therein), whereas measurements above terrestrial environments (e.g., wetlands, forests, and soils) are scarcer (12, 45, 46, 47). Large regional- to continental-scale data sets of total Se concentrations in soil as well as environmental variables (e.g., climate and soil physicochemical properties) have been used to identify drivers of Se concentrations in soil in top-down approaches (references 43 and 48 and references therein). Bottom-up and top-down approaches have shown considerable differences (a factor of ∼2 in Se fluxes) in global atmospheric emissions (43). Uncertainty in Se deposition maps in top-down approaches mainly arose from uncertainties in global emission fluxes because of the high variability of past flux measurements (48). Furthermore, the spatial distribution of terrestrial Se emissions is largely unknown (43).

The current study shows that S status—particularly Cy—has a decisive effect on the production of methylated, volatile Se species in aerobic conditions. Generally, thiols originate from microbial metabolism, the biotic and abiotic degradation of natural organic matter (NOM), the addition of sulfide to unsaturated organic compounds, and the release from anthropogenic activities (reference 49 and references therein). There are limited data on the concentrations of free Cy in the environment. Whereas it appears to be less concentrated (up to 34 nM) in boreal wetland porewater (49), cysteine concentration can be as high as 2.6 μM in epibenthic biofilms (50). Therefore, the question remains to what degree Se will be shuttled from methylated, volatile species to nonvolatile 2H3MSePA (and others) considering the higher (33 μM) concentrations used here. However, particularly in bioremediation and wastewater treatment systems, the often-high level of (reduced) S (for example, see references 28, 51, and 52) may limit the volatilization which has been suggested to reduce dissolved Se concentrations. Se is known to be heterogeneously distributed in soils at a microscopic to regional scale (for example, see references 3 and 53 and references therein). This study showed the influence of sulfur amino acids on methylation under aerobic conditions in pure cultures. Under more reducing conditions, dissimilatory reduction of selenate may be dominant (and thus elemental selenium and/or selenides, representing a sink for Se, limiting its bioavailability). However, if one assumes that in aerobic soils sulfur amino acids occur to some extent and that they are heterogeneously distributed, Se biomethylation may also be spatially highly variable in such soils. It remains to be elucidated whether this link of Se and S cycles exists in the environment in general and what role S speciation plays in particular.

Despite delivering measured (and not modeled) data, the disadvantages of rain/soil measurements include uncertainties in spatial and temporal variation, not to mention the laborious work of sample collection, analysis, and data processing. Therefore, transport models based on direct measurements of atmospheric samples may be more accurate if supplied with systematic (laboratory) data on volatilization efficiencies in different environmental conditions. The current study points to the need to further detail the interconnection of S and Se cycling. Here, DMDSe and DMSeS were the only species found. The first global Se deposition model, however, considered only DMSe, as it is usually the dominant emitted volatile Se compound (48). Identifying appropriate proxies of the S status of soils (e.g., total S, S oxyanions, and thiols) that correlate with Se volatilization to certain species (DMSe, DMDSe, and DMSeS) may increase the accuracy of global-scale distribution modeling in terms of the underpredictions still found (54). This is particularly important in view of Se deficiency possibly increasing with global warming, with consequences for the health of organisms (54).

## MATERIALS AND METHODS

### Chemicals.

All the media and aqueous solutions were prepared using ultrapure water (18.2 Ω·cm; Thermo Scientific, Nanopure, Reinach, Switzerland). Dimethylsulfide (DMS), dimethyl disulfide (DMDS), dimethyl selenide (DMSe), dimethyl diselenide (DMDSe), dimethyl trisulfide (DMTS), sodium selenite, sodium selenate, methylselenic acid, methionine (Met) and cystine (Cy) were purchased from Sigma-Aldrich (Buchs, Switzerland) and were of high purity (>99%). The later-identified compound designated unknown1 (2-hydroxy-3-(methylselanyl)-propanoic acid) was purchased from Tetrahedron (Paris, France) and was of ≥98% purity. The concentrations of the Se stock solutions were verified by triple-quadrupole inductively coupled plasma mass spectrometry (QqQ-ICP-MS) (see below). A minor impurity of selenate (∼7% or ∼0.46 μM Se in incubations) was found in the sodium selenite salt.

### Bacterial cultivation.

Pseudomonas tolaasii was obtained from DSMZ (Leibniz Institute German Collection of Microorganisms and Cell Cultures) (strain 19342). The strain was recovered in King’s B medium (10 g/liter peptone from casein, 1.5 g/liter K_2_HPO_4_, 15 g/liter glycerol, 5 ml/liter of 1 M MgSO_4_) and then transferred to King’s B medium agar plates (28°C). Precultures were prepared from single colonies in glutamine glucose minimum medium (GGM) (28°C, 180 rpm, 18 h) (55). The experimental media were prepared by spiking GGM with 500 μg Se/liter as selenite (= 6.33 μM Se) and S amino acids l-methionine (Met) and/or l-cystine (Cy) (33.3 μM each). Controls did not contain S amino acids. Subsequently, the spiked media were inoculated with precultures (2% vol), and 5 ml was distributed into headspace vials (20 ml). These were sealed with gas-tight polytetrafluoroethylene (PTFE)/silicone septa and stainless-steel screw caps. Samples were incubated at 28°C and 180 rpm. At each time point, headspace vials were sacrificed for sampling. Volatile methylated species were quantified by SPME-GC-MS (see below), and incubations were stopped by centrifugation (4,000 × *g*, 20 min) and quick-freezing (−20°C). For IC-QqQ-ICP-MS measurements, the supernatants were first filtrated by a syringe filter (0.8 μm, mixed cellulose ester), followed by vacuum pump filtration (0.22 μm). For the preconcentration and further identification of unknown1 (see below), 500 ml of experimental medium in 2-liter Schott bottles were inoculated with 10 ml of *P. tolaasii* precultures and incubated at 28°C and 180 rpm for 18 h.

### Quantification of methylated Se species.

DMS, DMDS, DMSe, DMDSe, DMTS, and DMSeS were quantified by SPME-GC-MS.

An Agilent 7890A GC coupled to a 5977 series mass selective detector equipped with a PAL autosampler system and automatic solid-phase microextraction (SPME) was used for measurements. Volatile species were sampled using a 75-μm Carboxen/polydimethylsiloxane (CAR/PDMS) SPME fiber (23 Ga; Supelco, Bellefonte, PA, USA) 1 cm in length. Details are given in the supplemental material (Table S1 and Fig. S1). Individual stock solutions of DMS, DMDS, DMSe, DMDSe, and DMTS were diluted with methanol (high-performance liquid chromatography [HPLC] grade, ≥99.9%) to 100 mg/liter. From these solutions, calibration solutions were made by dilution in ultrapure water in headspace vials (amber, 20 ml) closed by a magnetic screw cap and PTFE/silicone septum. DMSeS was also detected in bacterial culture supernatant. Pure DMSeS cannot be isolated in solution due to dynamic Se/S exchange reactions between DMDS and DMDSe. Therefore, we prepared DMSeS by mixing standard solution containing DMDS and DMDSe (56, 57). To quantify DMSeS, from these mixed standards, both remaining DMDS and DMDSe were quantified by using their respective single-compound calibration lines, yielding the concentrations for DMSeS according to DMDS + DMDSe ⇌ 2 DMSeS. From these calculated concentrations, the calibration line for DMSeS was established (i.e., area versus concentration calculated).

### Quantification of Se oxyanions and chromatographic separation of the unknown metabolites.

Se oxyanions (selenite and selenate) were separated from methaneseleninic acid using a Dionex 2100 ion chromatograph (IC) (Thermo Fischer, Reinach, Switzerland), as previously described (58). The IC was coupled to an Agilent 8800 QqQ-ICP-MS. Analytes were separated using a guard column (IonPac AG15; 2 × 50 mmol/liter) and an analytical column (IonPac AS15; 2 × 250 mmol/liter) (see the supplemental material). Peak fractions containing the unknown species were manually collected at the IC outlet after having passed the conductivity cell. The QqQ-ICP-MS was operated in mass-shift mode using O_2_ as a reaction gas (see Table S2). Se was measured as ^78/80^Se^16^O^+^ in time-resolved analysis.

### Concentration of the unknown Se metabolites.

Solid-phase extraction (SPE) was performed on an Oasis HLB 3-cm^3^ cartridge (Waters, Milford, MA, USA) previously conditioned with methanol (HPLC grade, ≥99.9% purity; Sigma-Aldrich) and ultrapure water (both 1 ml). After conditioning, filtrated (0.22 μm) supernatant was acidified to pH 3 (∼70 μl concentrated hydrochloric acid [HCl]; semiconductor grade; Sigma-Aldrich), and 15 ml was passed through the cartridge at ambient temperature and pressure. The cartridge was washed once by quickly passing it through 1 ml of 5% methanol in 1 mmol/liter formic acid (LC-MS grade; Sigma-Aldrich). After a washing, the Se species retained on the cartridge were eluted slowly with 0.5 ml 1.6% NH_3_·H_2_O (ACS reagent, 28.0 to 30.0% NH_3_ basis; Sigma-Aldrich). The SPE procedure was repeated to collect sufficient amounts of compounds for further analyses.

### Mass-spectrometric characterization of unknown Se metabolites.

An Agilent 6320 ESI-ion trap-MS was operated in the negative-ion mode. The fraction collected from IC containing the unknowns was directly introduced into the electrospray source using a syringe pump (KDS 100CE; KD Scientific, Holliston, MA, USA) (see the supplemental material). Unknown Se metabolites were recognized by the Se isotopic pattern in full-scan mass spectra. High-resolution MS was done on an Agilent 6540 UHD mass spectrometer equipped with Agilent Jet Stream ESI and operated in negative mode (see Table S3). The collected peak fraction of unknown1 was introduced into the ESI using the syringe pump at a speed of 5 μl/min. Data were processed by Agilent MassHunter Data Acquisition for 6500 series Q-TOF-MS (B.08.00). The possible chemical structures of unknown1 were generated based on accurate mass, isotope abundance, and isotope patterns using the “formula calculator” tool in Agilent MassHunter Qualitative Analysis (B.06.00 SP 1) software.

### Microprobe NMR characterization of unknown Se metabolites.

For microprobe NMR analysis, pooled IC fractions of unknown1 were lyophilized until dry. The white powder was then taken up in deuterium oxide (D_2_O; 10 μl). NMR spectra were recorded on a Bruker Avance III NMR spectrometer operating at 500.13 MHz for ^1^H nuclei. ^1^H-NMR and correlated spectroscopy (COSY) spectra were measured at 291 K in a 1-mm TXI probe with a z-gradient. ^1^H-NMR spectra were referenced on the residual proton signal of the solvent [D_2_O: δ(^1^H) = 4.79 ppm].
