# Supplementary material for: Sulfur Amino Acid Status Controls Selenium Methylation in Pseudomonas tolaasii: Identification of a Novel Metabolite from Promiscuous Enzyme Reactions
Source: Appl Environ Microbiol. 2021 May 26;87(12):e00104-21. doi: 10.1128/AEM.00104-21 (PMC8174768; doi:10.1128/AEM.00104-21)
Supplement: SUPPLEMENTAL FILE 1 — Supplemental material. Download aem.00104-21-s0001.pdf, PDF file, 1.1 MB [file aem.00104-21-s0001.pdf]

Supplementary Information to Sulfur amino acids status controls selenium methylation in *Pseudomonas tolaasii*: Identification of a novel metabolite from promiscuous enzyme reactions

Ying Liu[a,b]; Sebastian Hedwig[a],[c]; Andreas Schäffer[b]; Markus Lenz[a,d*]; Mathieu Martinez[a]

[a] Institute for Ecopreneurship, School of Life Sciences, University of Applied Sciences and Arts Northwestern Switzerland, Hofackerstrasse 30, 4132 Muttenz, Switzerland

[b] Institute for Environmental Research, RWTH Aachen University, Worringerweg 1, 52074 Aachen, Germany

[c] University of Basel, Department of Chemistry, Mattenstrasse 24a, 4058 Basel, Switzerland,

[d] Sub-Department of Environmental Technology, Wageningen University, 6700 AA, Wageningen, the Netherlands

*Correspondence: markus.lenz@fhnw.ch

a. Quantification of methylated Se species by SPME-GC-MS

Any new SPME fiber was conditioned at 300 °C under the N_2 flow for 30 min. After each measurement, the fibers were cleaned by heating in 300 °C for 3 min. The extractions were as follow: 1 min of vial preheating and 30 min of extraction with agitation at 250 (both at 45 °C); desorption to GC injector at 300 °C for 4 min.

Table SI1: SPME-GC-MS parameters

Column	Agilent DB-5; 60 m × 0.32 mm ID; 0.25 µm coating
Purge time	0.5 min He min^{-1}
Purge flow	3 mL min^{-1}
Injector temperature	300 °C, splitless
Column temperature program	4 min holding time at 35 °C heating with 7 °C min^{-1} to 140 °C, heating at 25 °C min^{-1} to 280 °C with 1 min hold time at 280 °C total time 25.6 min
Carrier gas	1 mL He min^{-1}
GC-MS transfer liner temperature	280 °C
MS Source temperature	230 °C
MS Quad temperature	180 °C
EI energy	70 eV
SIM target ions (m/z)	62 (DMS), 110 (DMSe), 94 (DMDS), 142 (DMSeS), 190 (DMDSe), 126 (DMTS)
SCAN mass range (m/z)	45–250

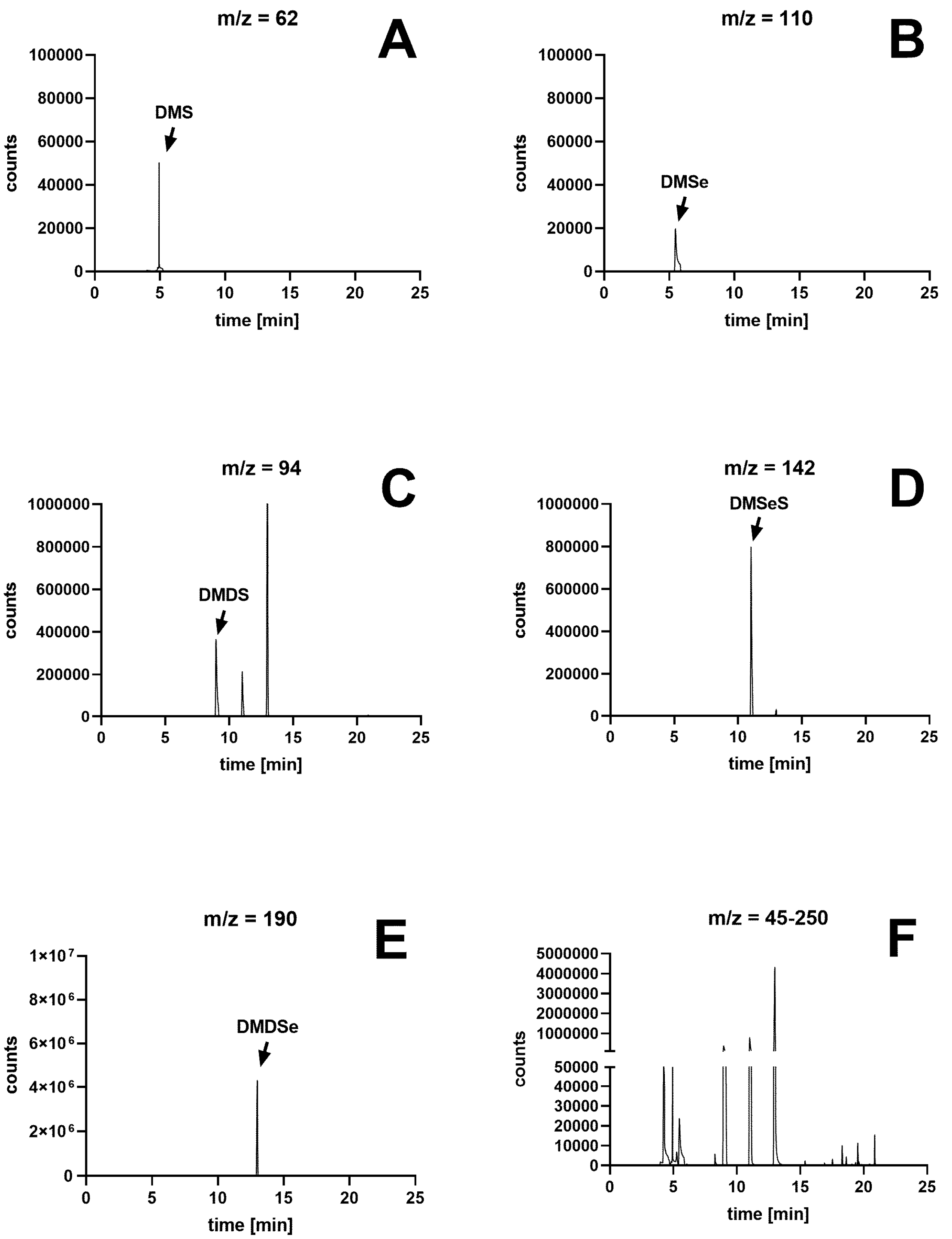

Figure SI1: Exemplary separation of methylated species in a culture supernantant by SPME-GC-MS.

b. Quantification of Se oxyanions and chromatographic separation of the unknown metabolites by IC-QqQ-ICP-MS

The separation was done at 35 °C and a flow of 0.5 mL/min. The KOH gradient was as follows: 0–4.4 min at 40 mmol/L; 4.4–4.5 min increase to 45 mmol/L; from 4.5–7 min linearly increase to 65 mmol/L; from 7–9.9 min hold at 65 mmol/L; 9.9–10 min decrease to 40 mmol/L; 10–11 min hold at 40 mmol/L. The injection volume was 25 µL.

Table SI2: ICP-QQQ-MS operating conditions.

Nebulizer	MicroMist 0.4 mL min^{-1}
Spray chamber & torch	Scott type, quartz
Cones	Ni
Internal standard	0.1 ppm Rh in 2% HNO_3
Plasma flow rate (L min^{-1})	15
Plasma power (W)	1550
Carrier gas flow (L min^{-1})	0.99
Peri pump (rps)	0.4
S/C temp (°C)	2
Oxygen flow rate (%)	30
Detected ions	$^{80}SeO^+$, $^{78}SeO^+$
Q1 m/z	78, 80
Q2 m/z	94, 96
Integration time (msec)	?
Wait Time Offset (msec)	2

c. Mass spectrometric characterization of unknown Se metabolites by ESI-ion trap-MS and Q-TOF-MS

For ESI-ion trap-MS, samples were delivered at a speed of 5 µL min^{-1}. Parent ions were scanned within range between m/z 50 to 500 with a maximum accumulation time of 200 ms. Capillary spray voltage was set to +1500 V. Helium (He) was used as a nebulizing gas at a pressure of 10 psi and as drying gas at a flow rate of 5 L min^{-1} (drying temperature, 325 °C). Fragments of ions were generated with amplitude 0.5 V and He as a collision gas, with a precursor ion isolation width of 2 amu.

Table SI3: Q-TOF-MS operating conditions.

Nebulizer pressure (psig)	20	Nozzle voltage (V)	2000
Sheath Gas Flow (L min^{-1})	6	Sheath Gas Temperature (°C)	250
Drying Gas Flow (L min^{-1})	5	Drying Gas Temperature (°C)	250
Capillary voltage (V)	3000	Fragmentor voltage (V)	175
Skimmer (V)	65	OCT1RFVpp (V)	750
Instrument mode		High resolution mode, 4 GHz	

[M − H]⁻: 182.95659 Da   Molecular Formula: $C_4H_8O_3Se$

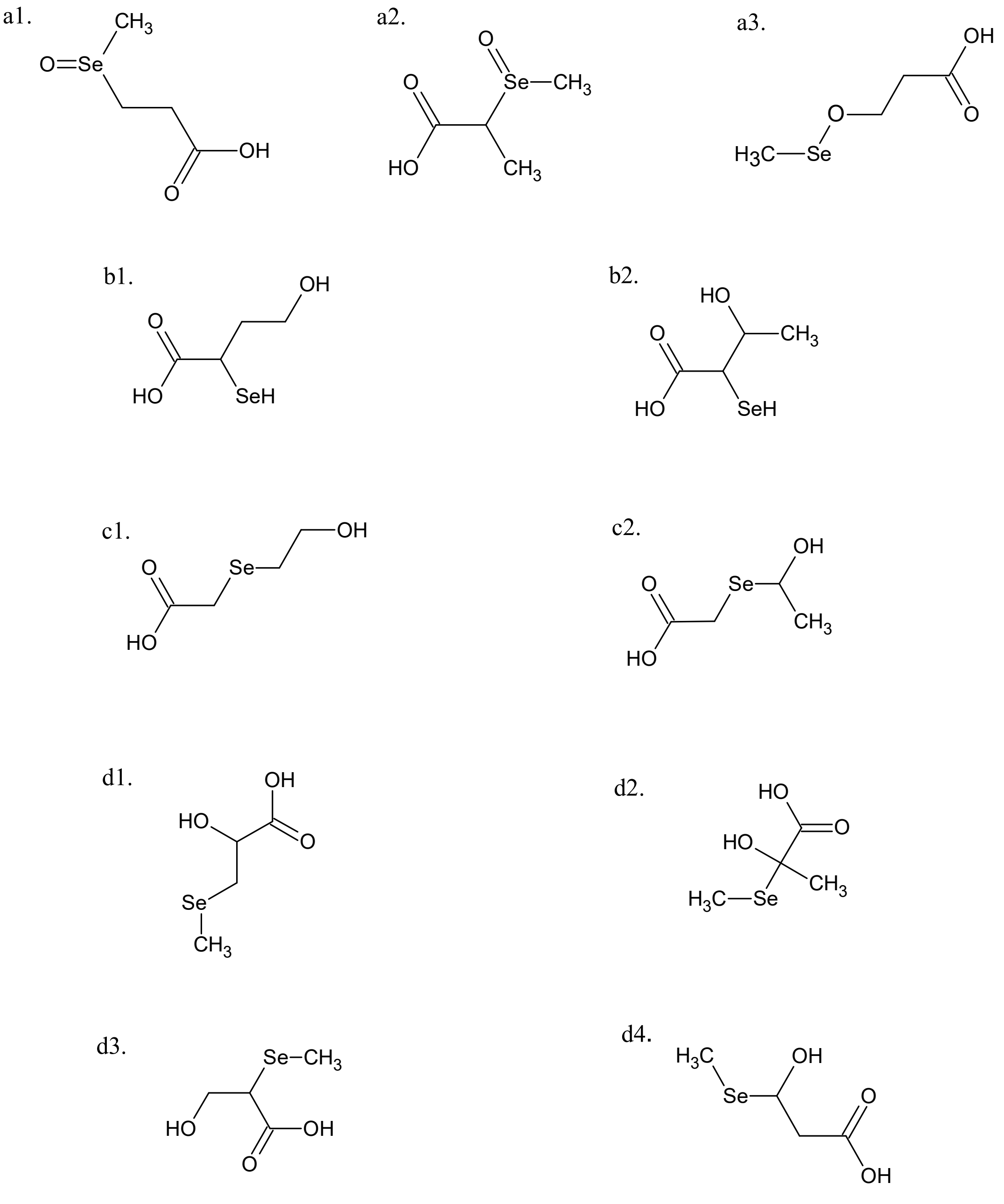

Figure SI2: Candidates for the unknowns of $C_4H_8O_3Se$ based on the exact mass determined by Q-Tof-MS.

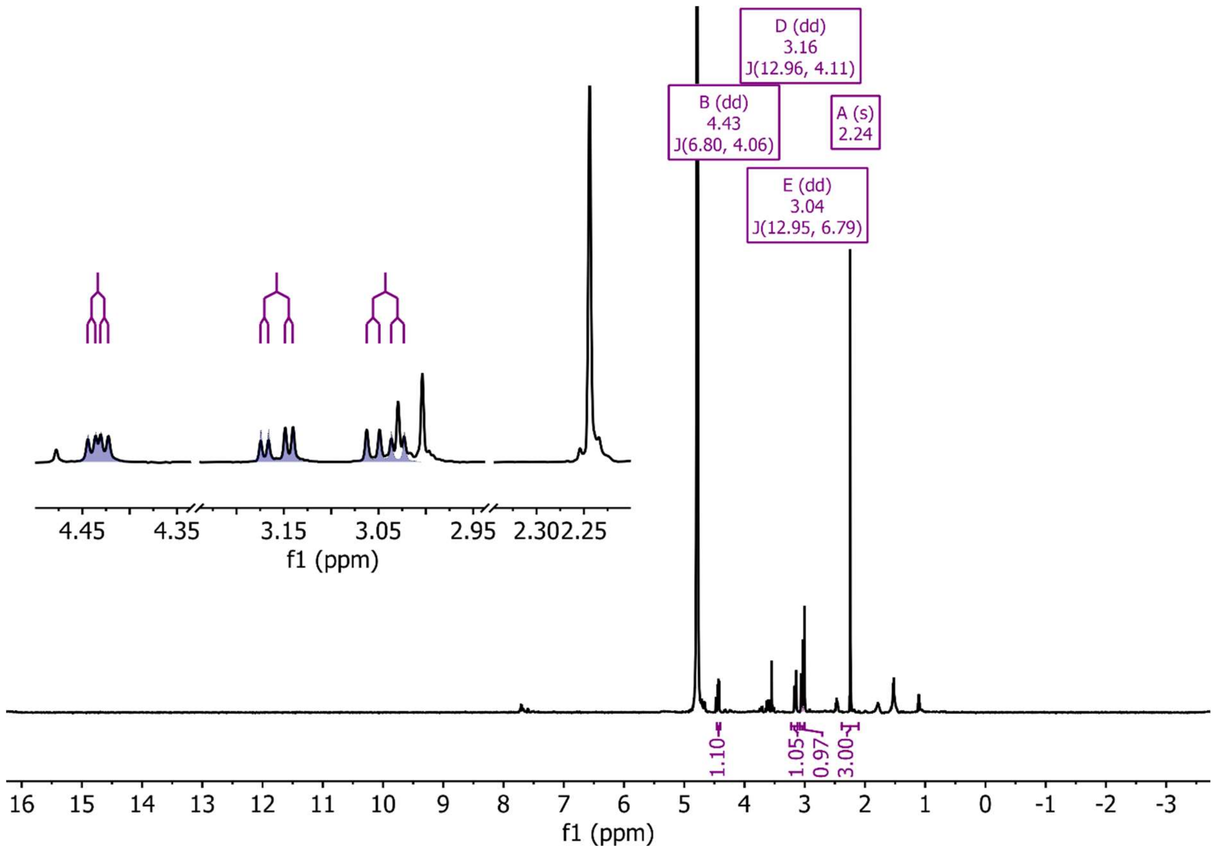

Figure SI3: ^{1}H-NMR spectrum of the lyophilizate of unkown1 dissolved in D_2O (293 K, 500 MHz). Multiplets belonging to proton signals of unkown1 were highlighted in the expansion for the discrimination of impurity peaks.

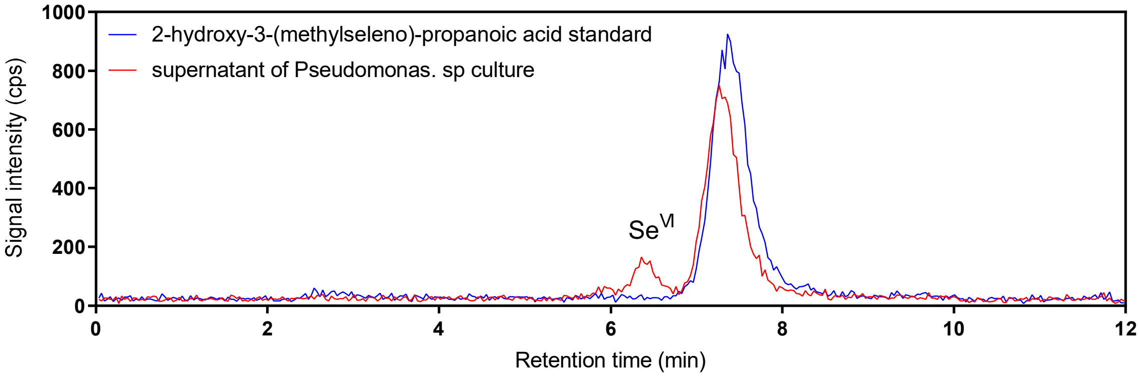

Figure SI4 Separation of selenium species by IC-ICP-QQQ (mass shift mode using signal $^{78}Se^{16}O^{+}$) of unkown1 and 2-hydroxy-3-(methylselanyl)propanoic acid) in *Pseudomonas* culture medium.

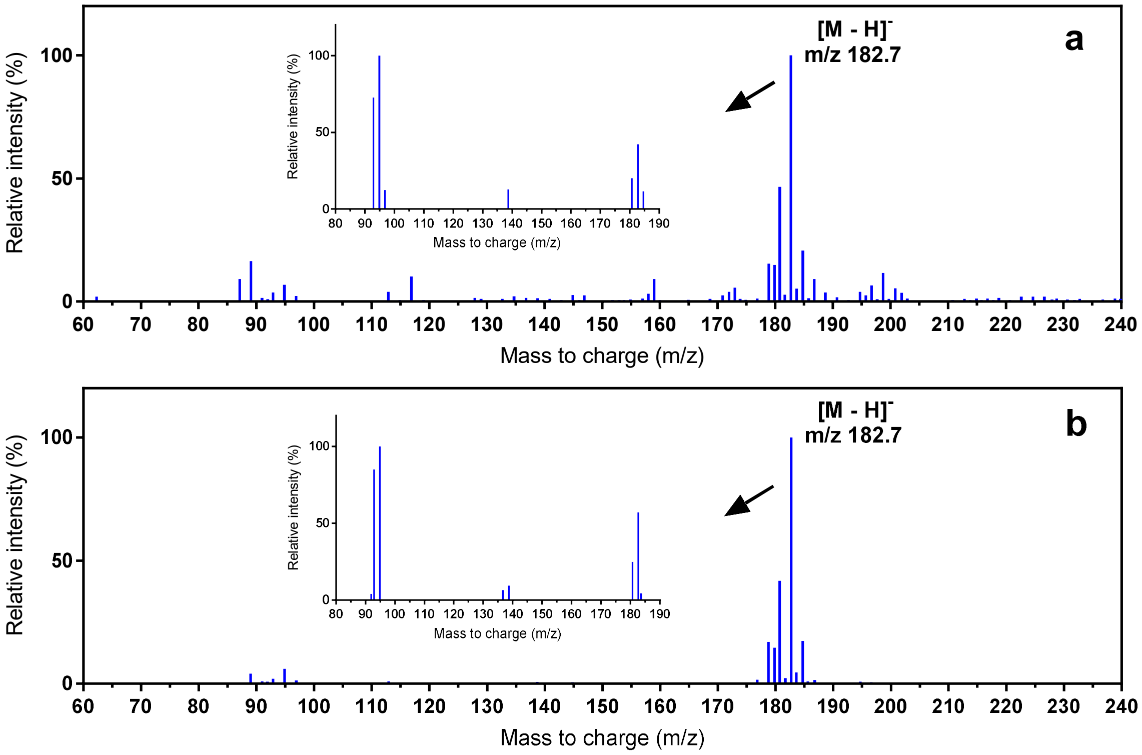

Figure SI5 Mass spectra and MS/MS spectra of $[M{-}H]^{-}$ obtained from helium collision-induced fragmentation of (a) the collected fraction corresponding to unknown1 and (b) standard of 2-hydroxy-3-(methylselanyl)propanoic acid in ultrapure water.
